# Molecular Detection of Southern Tomato Amalgavirus Prevalent in Tomatoes and Its Genomic Characterization with Global Evolutionary Dynamics

**DOI:** 10.3390/v14112481

**Published:** 2022-11-09

**Authors:** Muhammad Dilshad Hussain, Tahir Farooq, Xi Chen, Tong Jiang, Lianyi Zang, Muhammad Taimoor Shakeel, Tao Zhou

**Affiliations:** 1 State Key Laboratory for Agro-Biotechnology, and Ministry of Agriculture and Rural Affairs, Key Laboratory for Pest Monitoring and Green Management, Department of Plant Pathology, China Agricultural University, Beijing 100193, China; 2 Plant Protection Research Institute and Guangdong Provincial Key Laboratory of High Technology for Plant Protection, Guangdong Academy of Agricultural Sciences, Guangzhou 510640, China; 3 College of Plant Protection, Collaborative Innovation Center of Fruit & Vegetable Quality and Efficient Production in Shandong, Shandong Agricultural University, Tai’an 271018, China; 4 Department of Plant Pathology, Faculty of Agriculture & Environment, The Islamia University of Bahawalpur, Bahawalpur 63100, Pakistan

**Keywords:** southern tomato amalgavirus, cryptic pathogen, vertical transmission, viral prevalence, next-generation sequencing, phylodynamics, genetic variability

## Abstract

Southern tomato amalgavirus (STV) is a cryptic pathogen that is abundant in tomato production fields and intensifies the resurgence of tomato yellow stunt disease (ToYSD), together with other phytoviruses. Here, we mapped the geographical and genomic diversity, phylogenetics, and evolutionary dynamics of STV. We found that STV prevailed across China and Pakistan, with a maximum average rate of infection of 43.19% in Beijing, China, and 40.08% in Punjab, Pakistan. Subsequently, we amplified, cloned, and annotated the complete genome sequences of STV isolates from *Solanum lycopersicum* L. in China (OP548653 and OP548652) and Pakistan (MT066231) using Sanger and next-generation sequencing (NGS). These STV isolates displayed close evolutionary relationships with others from Asia, America, and Europe. Whole-genome-based molecular diversity analysis showed that STV populations had 33 haplotypes with a gene diversity (Hd) of 0.977 and a nucleotide diversity (π) of 0.00404. The genetic variability of RNA-dependent RNA-polymerase (RdRp) was higher than that of the putative coat protein (CP) p42. Further analysis revealed that STV isolates were likely to be recombinant but with a lower-to-moderate level of confidence. With a variable distribution pattern of positively and negatively selected sites, negative selection pressure predominantly acted on p42 and RdRp. These findings elaborated on the molecular variability and evolutionary trends among STV populations across major tomato-producing regions of the world.

## 1. Introduction

**Figure 1:**
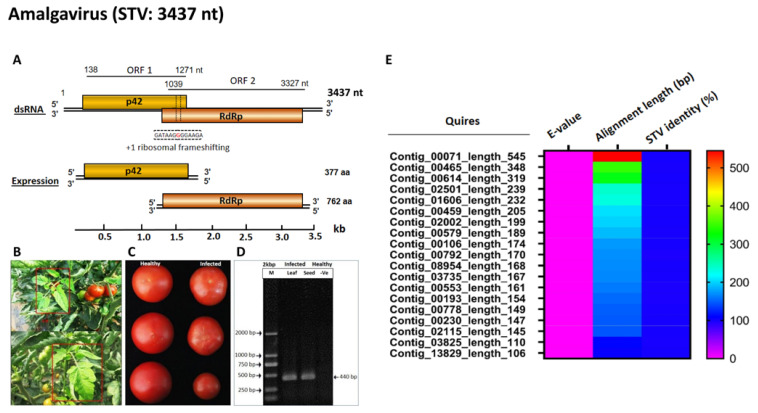
The genomic organization and detection of STV through reverse transcription–polymerase chain reaction (RT-PCR) from infected tomato leaf and seed samples. (**A**) Schematic representation of the genome organization and translation of STV, including the UTR regions and gene distribution. The nucleotide positions of open reading frames (ORFs) were exemplified using the STV Pk isolate (MT066231). (**B**) Infected plants displaying typical chlorotic, leaf yellowing (shown in red boxes), stunting, and fruit malformation. (**C**) Tomato fruit with discoloration and size reduction alongside fruit from healthy tomato plants. (**D**) Agarose gel electrophoresis of RT-PCR products indicating amplified STV fragments that covered a conserved region of p42 and RdRp at the position of ribosomal frameshifting from infected tomato leaf and seed samples compared with a healthy sample. (**E**) Heatmap columns representing different parameters (E-value, alignment length, and percentage identity) associated with contigs mapped to the STV genome. Pink and red colors denote the lowest and highest values, respectively.

Plant viral diseases are major impediments to the sustainable food production system across the world [1,2,3,4,5]. In particular, the production of tomatoes (*Solanum lycopersicum* L.) is under persistent constraint due to innumerable viruses [6,7]. Southern tomato amalgavirus (STV) belongs to the genus *Amalgavirus* in the family *Amalgaviridae*, consisting of a double-stranded RNA (dsRNA) genome of 3.5 kb that encodes two proteins from two overlapping open-reading frames (ORFs): ORF 1 encodes for the putative coat protein (CP) p42 and ORF 2 encodes for the RNA-dependent RNA-polymerase (RdRp), a fusion protein that is expressed by a +1 ribosomal frameshift (Figure 1A). STV is a persistent quarantine pathogen that is transmitted vertically through tomato seeds, while horizontal transmission has not been reported [8]. STV infects tomato lines and varieties in general because it accompanies the embryo seed. It is referred to as a “cryptic plant virus”, meaning that it occurs in plants without showing obvious symptomology under a single infection condition. However, in association with other viruses, including tomato yellow leaf curl begomovirus (TYLCV), tomato chlorosis crinivirus (ToCV), tomato infectious chlorosis crinivirus (TICV), pepino mosaic potexvirus (PepMV), cucumber mosaic cucumovirus (CMV), tomato mosaic tobamovirus (ToMV), and tomato spotted wilt orthotospovirus (TSWV), STV exhibits chlorotic, leaf yellowing, stunting, and fruit malformation with the induction of tomato yellow stunt disease (ToYSD) (Figure 1B,C) [8,9,10,11,12,13].

STV may be prevalent all over the world, but its identification remains challenging due to the absence of symptoms in single infections. Thus, high-throughput sequencing (HTS) technology, such as next-generation sequencing (NGS), has been employed to detect STV in many countries, including the United States of America, Spain, Germany, Italy, China, Bangladesh, and Pakistan [10,14,15,16,17]. During the infection, STV interacts with the host or/and with other associated partner viruses and demonstrates evolutionary dynamics/variability. Transcriptome analysis revealed that STV modifies the expression of endogenous plant micro-RNAs (miRNAs) and virus-derived small interfering RNAs (vsiRNAs) in tomato plants [9]. For example, in tomato plants with a singular infection of STV, the amount of plant-miRNAs is increased and they are involved in the regulation of several complex cellular pathways against pathogens, while the amount of STV-vsiRNAs are decreased without the induction of symptoms and cellular ultra-structural modification, but increased in the presence of CMV and PepMV with additional symptomologies [9,18,19]. Furthermore, STV infection was also reported to abrogate the antagonism between CMV and PepMV, reinstating the CMV titer and viral symptoms [18].

Over the last decade, metagenomic studies through HTS demonstrated that plants are frequently infected by an unprecedented number of persistent viruses, increasing the nucleotide datasets for determining the genomic characteristics of these pathogens [20,21,22]. To investigate STV infection, we conducted disease inspections in China and Pakistan and detected STV in single and mixed infections with other suspected viruses from symptomatic tomato plants and their seeds through reverse transcription–polymerase chain reaction (RT-PCR) and NGS. Strikingly, NGS confirmed the presence of STV with some additional previously reported pathogenic viruses, including ToCV, TYLCV, TICV, and ToMV in tomato plants, which induce symptoms that are identical to those observed in collected samples. In addition, the infection of STV was also validated via RT-PCR using specific pairs of primers and Sanger sequencing [8]. The complete the genome sequences of STV, isolates from Pakistan and China were obtained. To date, no study has comprehensively analyzed the genetic diversity and evolvability among global populations of STV. Therefore, we also analyzed the genetic diversity and evolutionary dynamics of STV, which will assist in understanding the genetic complexity and evolution of this virus that are presumably governing the continual STV global spread and successful adaptation to different ecosystems. Furthermore, these findings are imperative for understanding the epidemiology of STV and developing effective detection methods and control strategies.

## 2. Materials and Methods

### 2.1. STV Infection Status and Samples Collection 

**Figure 2:**
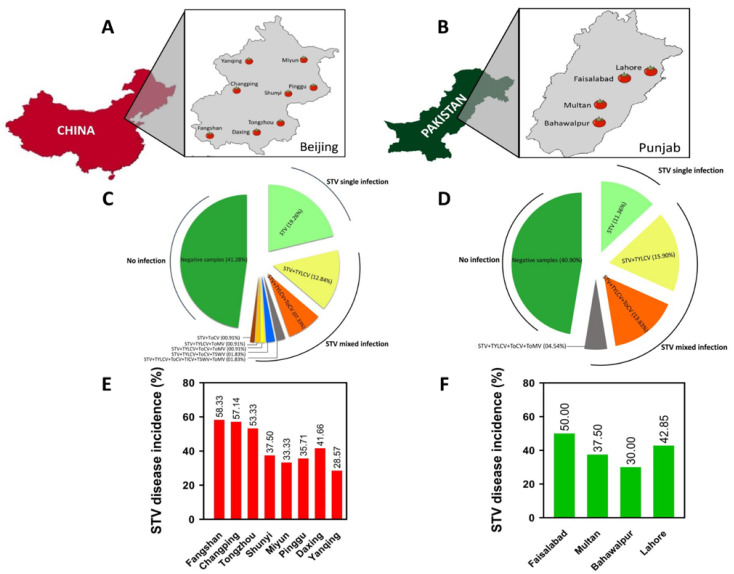
The geographical studies of STV with its diagnosis and infection dynamics. (**A**,**B**) The geographical locations of disease-symptomatic tomato samples collected from Beijing, China, and Punjab, Pakistan. Sampled sites are marked on the enlarged abridged maps. (**C**,**D**) The infection dynamics of STV in single- and mixed-infection conditions with multiple combinations of viruses. (**E**,**F**) The confirmed STV disease incidence recorded across Beijing, China (**E**), and Punjab, Pakistan (**F**).

To investigate the STV infections, characteristic symptoms of ToYSD, such as pronounced chlorotic, leaf yellowing, and stunting symptoms were examined on tomato plants with discoloration, deformed, and reduced fruit size under field, green-, and glass-house conditions. Symptomatic tomato plants were indexed for the presence of the following viruses: TYLCV [23], ToCV [24], TICV [25], and STV in single- and mixed-infection conditions [8]. Tomato leaf and seed samples used in this study were collected from multiple field visits at different localities in Beijing, China, and Punjab, Pakistan, from 2017 to 2020 (Figure 2A,B and Table S1)**.** These samples were subjected to laboratory analyses for the molecular detection and confirmation of STV infection due to single and multiple viruses. The rate of STV infection and ToYSD prevalence and incidence were recorded using previously described formulas [26] (Table S1)**.** In addition, harvested leaf samples were also stored in sterilized pre-cold 2 mL Eppendorf safe-lock microcentrifuge tubes at −80 °C for total RNA preparation and subsequent experimentations.

### 2.2. Total RNA Preparation and Virus Detection

Total RNA was extracted from 100 mg of freeze-dried leaf and seed material from each of the individual samples employed in this study using TRIzol reagent (Invitrogen, Life Technologies, Carlsbad, CA, USA) following the manufacturer’s instructions. The quality and quantity of RNA were determined spectrophotometrically at a 280 nm wavelength with a NanoDrop spectrophotometer (ND-1000, Fisher Thermo, Wilmington, DE, USA). Subsequently, RNA aliquots of smaller volumes per sample were stored at −80 °C until further analysis. 

### 2.3. Detection Using RT-PCR

To test the infection of STV and its associated viruses, as well as to validate the STV infection rate across different localities using RT-PCR, these extracted RNAs were reverse-transcribed into complementary DNAs (cDNAs) with the Moloney murine leukemia virus–reverse transcriptase (MMuLV-RT) (Sigma Aldrich, St. Louis, MO, USA) in a total volume of 20 μL per sample. These cDNAs were subjected to PCR with a *Taq* DNA polymerase (Sigma Aldrich, USA) using virus-specific pairs of primers (Table S2) [8,27,28,29,30,31,32,33,34]. PCR assays were performed for the amplification of target fragments of each suspected virus. Amplified PCR products were analyzed using gel electrophoresis on 1% agarose gels alongside a 1 kb DNA ladder (Thermo Fisher Scientific, Waltham, MA, USA) prestained with ethidium bromide (10 mg/mL) in 1× Tris-acetate-EDTA (TAE) buffer, pH 8.0. The gels were visualized with the help of the Gel Doc XR imaging system (Bio-Rad, Hercules, CA, USA). Positive RT-PCR products were gel-purified and sequenced directly to confirm the presence of the detected virus [29,35].

### 2.4. Detection Using NGS 

Samples from high-STV-incident regions were subjected to NGS for detection in single and or mixed infections with a range of plant viruses. Total extracted RNA was purified by adding 0.1 volume of NH_4_OAc (Sigma Aldrich, USA) and 2.5 volumes of 100% pre-cold EtOH (Sigma Aldrich, USA) and the RNA was directly incubated at −80 °C for 30 min. After that, the RNA was incubated at room temperature for 5 min and centrifuged for 20 min at 12,000 RPM. Subsequently, the pellet was washed with 75% pre-cold EtOH (Sigma Aldrich, USA) and re-suspended in RNase-free H_2_O [36,37]. The purity and concentration of the RNA were determined with a NanoDrop spectrophotometer. Furthermore, to test the quality and integrity of the purified total RNA using gel electrophoresis, an aliquot of RNA (500–1000 ng) alongside a 5 kb DNA ladder (Thermo Fisher Scientific) was run on 1% agarose gel stained with ethidium bromide for ~30–60 min. The RNA bands of 28/23S rRNA and 18/16S rRNA were clearly visualized, and the brightness of 28/23S rRNA was greater than that of 18/16S rRNA. RNA integrity number (RIN) values ≥ 8.0, OD260/280 ≥ 1.9, and OD260/230 ≥ 1.5 were established. This intact RNA was subjected to the Illumina Hiseq sequencing platform to complete small-RNA sequencing, and the Illumina SE library was constructed for HTS. HTS followed by the de novo assembly resulted in sequence contigs. The data were analyzed using the Basic Local Alignment Search Tool (BLAST) from the National Center of Biotechnology Information (NCBI) and bioinformatics methods for the identification of suspected viruses and mapping their genomic sequences [38,39,40,41]. The NGS results were also validated using RT-PCR. 

### 2.5. Amplification and Molecular Cloning of the STV Genome 

**Figure 3:**
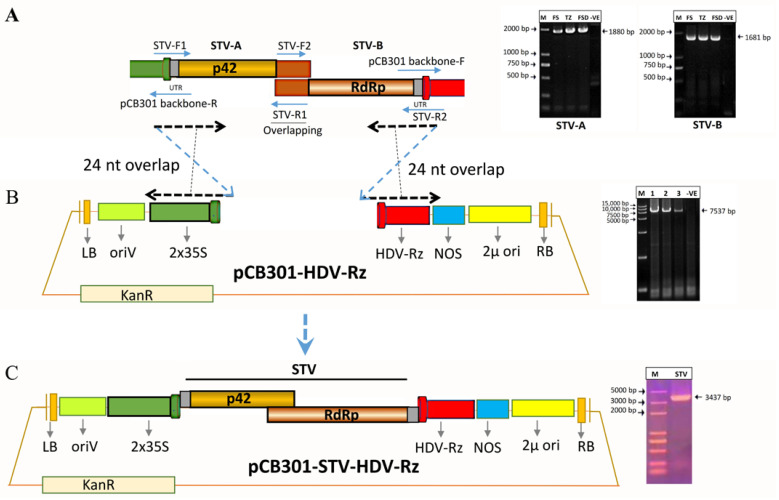
The schematic representation of the STV full-length genome and molecular cloning. (**A**) The STV full-length genome was amplified into two parts with overlapping pairs of primers (overlapping 24 nt from the 3′- and 5′-UTR of STV to the vector plasmid pCB301-2µ-HDV) with high-fidelity PCR. (**B**) The pCB301-HDV-Rz was linearized between the CaMV 35S promoter and the HDRz sequence using high-fidelity PCR. (**C**) Amplified STV parts A and B were assembled via yeast homologous recombination cloning and the full-length genome was constructed.

The STV dsRNA genome (3.5 kb) was amplified into two fragments, namely, STV-A (1880 bp) and STV-B (1681 bp), via high-fidelity PCR with Phusion high-fidelity polymerase (NEB, Ipswich, MA, USA) using two pairs of overlapping primers (STV-F1/STV-R1 and STV-F2/STV-R2) (Table S2), which were homologous to vector plasmid pCB301-2µ-HDV. The plasmid pCB301-2µ-HDV (7838 bp) was linearized between the CaMV 35S promoter and the HDRz sequence with high-fidelity PCR and amplified using a specific pair of primer (pCB301 backbone-F and pCB301 backbone-R) (Table S2), which was homologous to STV (Figure 3A,B). Yeast homologous recombination cloning was applied to assemble these purified DNA fragments of the viral genome in the yeast *Saccharomyces cerevisiae* through the pCB301-HDV-Rz vector to construct the full-length genome of the virus (Figure 3C) [42]. This strategy was adopted to amplify the full-length genome of STV from all other STV-positive samples, including tomato seed samples, and their genomes were submitted to GenBank^®^ (NCBI, Bethesda, MD, USA).

### 2.6. Multiple Sequence Alignment and Phylogenetics

The sequences of globally reported STV isolates were retrieved from the GenBank^®^ (NCBI, MD, USA) (Table S3). Multiple sequence alignments (MSAs) were executed based on the complete genome sequences of STV using the MUSCLE tool in the software Geneious Prime version 9.0.2. Likewise, alignments of the individual STV genes (p42 and RdRp) among the corresponding genes of the globally reported STV isolates were performed. All alignments were manually analyzed and adjusted (when necessary) before proceeding to the subsequent analysis. Phylogenetic analysis was performed with the molecular evolutionary genetics analysis computing platform using MEGA X [43,44]. The phylogenetic model was constructed with MEGA X employing the maximum likelihood (ML) method with 1000 bootstrap replicates [43]. The model was visualized and annotated using iToL [45]. Finally, the distribution and matrix of the pairwise identities among all STV isolates were determined using Sequence Demarcation Tool (SDT) v1.2 [46].

### 2.7. Estimation of the Nucleotide Diversity and Haplotype Variability Indices

The nucleotide diversity π (represented by the average pairwise number of nucleotide differences per site) was calculated using DnaSP V.5 [47]. The significant differences in the average nucleotide diversity among all STV sequences were estimated by calculating their 95% bootstrap confidence intervals. A 100 nt sliding window with a step size of 10 nt across the full-length sequences of STV was considered to calculate π. Additional population-genetics-related parameters, including the number of haplotypes (H), the haplotype diversity (Hd), the nucleotide diversity (π), the number of polymorphic sites (S), Watterson’s theta (θw), the total number of mutations (Eta), and Tajima’s D, were also estimated for STV genomes and individual coding sequences (p42 and RdRp) using DnaSP V.5 [48].

### 2.8. Recombination Analysis of STV Populations

The occurrence of recombination events across full-length STV sequences was investigated by using several methods, including Rdp, SisterScan, Bootscan, Chimaera, GeneConv, MaxChi, and 3Seq. The recombination analysis was implemented in the recombination detection program (RDP) V.4 [49]. For all methods, alignments were performed with default settings. The *p*-values less than the Bonferroni-corrected cutoff (0.05) were used to infer the statistically significant results. The recombination events detected by one, two, or three methods were regarded as events with low, moderate, or high levels of confidence, respectively.

### 2.9. Analysis of Positive and Negative Selection

The identification of potential positively and negatively selected sites in the coding sequences of p42 and RdRp was performed by using four distinct methods: single-likelihood ancestor counting (SLAC), partitioning for robust inference of selection, fixed-effects likelihood, and random-effects likelihood [50]. All these methods were employed in the adaptive evolutionary tool “Datamonkey”, which is available online at www.datamonkey.org (accessed on 27 April 2022) [51]. To exclude the possibility of misleading results, the recombination breakpoints among all STV sequences (p42 and RdRp) were searched by implementing the Genetic Algorithm Recombination Detection (GARD) method [52]. 

## 3. Results

### 3.1. STV Diagnosis and Infection Dynamics

STV diagnosis and infection rates were studied in all locations in Beijing, China, and Punjab, Pakistan, where tomato leaf and seed samples were collected based on the characteristic symptomology of ToYSD (Table S1 and Figure 2A,B). The most common symptoms were observed in tomato production fields, including pronounced chlorotic, leaf yellowing, and stunting symptoms on tomato plants with discoloration, deformed, and reduced fruit size under field, green-, and glass-house conditions. Interestingly, STV was detected in all tomato production regions and localities with ToYSD symptomology in both single- and mixed-infection conditions with a variable rate of infection (Table S1). Thus, ToYSD was most prevalent in the south of Beijing, such as Tongzhou (83.33%), Fangshan (80.00%), and Daxing (75.00%), whereas tomato production fields in the north of Beijing, including Miyun (60.00%), Yanqing (66.66%), and Shunyi (66.66%), except Changping (75.00%), as well as east of Beijing, namely, Pinggu (60.00%), which showed mild prevalence. Similarly, the east of Punjab, including Faisalabad (87.50%) and Lahore (80.00%), showed higher ToYSD prevalences compared with the south of Punjab, such as Bahawalpur (60.00%) and Multan (75.00%) (Table 1). Based on the laboratory diagnosis (PCR and sequencing analysis), STV incidence also varied from region to region, ranging from 28.57% in Yanqing (northwest of Beijing, China) to 58.33% in Fangshan (southwest of Beijing, China), and 30.00% in Bahawalpur (south of Punjab, Pakistan) to 50.00% in Faisalabad (east of Punjab, Pakistan), with an overall mean incidence of 43.19% for all eight districts of Beijing, China, and 40.08% for the four districts of Punjab, Pakistan, respectively (Table 1). Moreover, using RT-PCR, STV was detected in symptomatic tomato plants and their seed samples in single and mixed infections with other suspected viruses, such as TYLCV, ToCV, TICV, TSWV, and ToMV, in diverse combinations with different rates of infection (Figure 2C,D). NGS technology was also employed to detect STV and validate its infection dynamics under different combinations of viruses from the highest disease incidence regions, namely, Fangshan, Tongzhou, and Faisalabad. Together with RT-PCR, Sanger sequencing, and NGS studies, in Beijing, China, an infection rate of 19.26% STV was recorded in a single infection and 26.60% in mixed infections collectively with multiple viral infection combinations. Similarly, in Punjab, Pakistan, an incidence of 11.36% STV in single infections and 34.09% in mixed infections was observed (Figure 2C,D). Generally, STV causes severe infection in association with other viruses. In Beijing, severe infection was reported in Fangshan (58.33%), Changping (57.14%), and Tongzhou (53.33%), followed by Daxing (41.66%), Shunyi (37.50), Pinggu (35.71%), Miyun (33.33%), and Yanqing (28.57%). However, in Punjab, Faisalabad (50.00%) and Lahore (42.85%) had higher STV incidences than Multan (37.50%) and Bahawalpur (30.00%) (Figure 2E,F).

### 3.2. Amplification of the STV Genome and Molecular Cloning

STV encompasses a dsRNA (3.5 kb) genome with two dynamically functioning ORFs that overlap and encode two distinct proteins, such as ORF 1 encoding p42 (CP) and ORF 2 encoding RdRp via +1 ribosomal frameshifting. In order to perform STV genomic characterization and phylogenetic analysis, full-length genomes were constructed from the three highest virus incidence regions, namely, Fangshan, Tongzhou, and Faisalabad. The STV dsRNA genome, having two overlapping ORFs, was amplified into two fragments, namely, STV-A (1880 bp) and STV-B (1681 bp), through high-fidelity PCR using two pairs of primers (STV-F1/STV-R1 and STV-F2/STV-R2) (Supplementary Table S2), which were homologous to vector plasmid pCB301-2µ-HDV (Figure 3A). The plasmid pCB301-2µ-HDV (7838 bp) was linearized between the CaMV 35S promoter and the HDRz sequence with high-fidelity PCR using a specific pair of primers (pCB301 backbone-F and pCB301 backbone-R) (Supplementary Table S2) that was homologous to STV (Figure 3B). These STV-amplified fragments were assembled and cloned in the pCB301-HDV-Rz vector to construct the full-length genome of the virus through yeast homologous recombination cloning (Figure 3C) [42]. The whole genome was sequenced using Sanger sequencing technology. To have more genomic data for the execution of molecular characterization and phylogenetics from geographically different regions, STV full-length genomes were constructed from Fangshan, Tongzhou, and Faisalabad, and sequenced and deposited in GenBank^®^ (NCBI, USA) under the accession numbers OP548653, OP548652, and MT066231, respectively.

### 3.3. Multiple Sequence Alignment and Molecular Phylogenetics 

**Figure 4:**
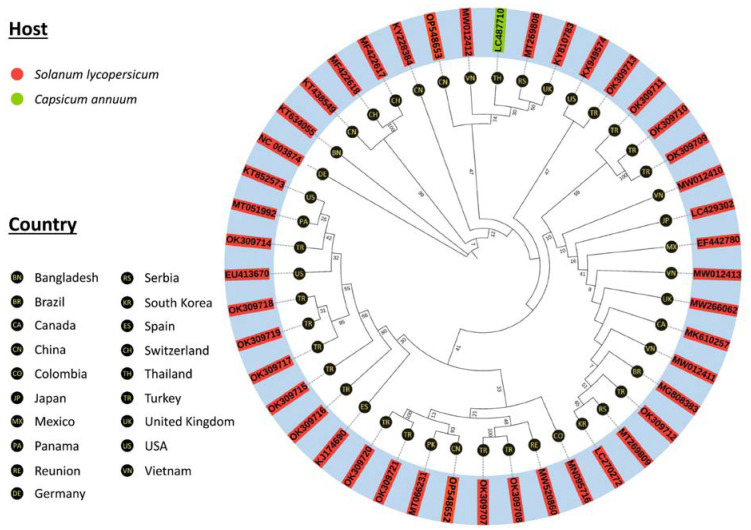
Molecular phylogenetics of STV demonstrated the evolutionary relatedness between the species of the genus *Amalgavirus* in the family *Amalgaviridae*. A phylogenetic radial tree was constructed on the basis of the complete genome sequence STV. The isolates are represented in the radial tree with accession numbers of the sequences and reported regions. All 44 STV isolates deposited in GenBank were used to analyze the phylogenetic relationships of isolates found in Beijing, China (OP548653 and OP548652), and Punjab, Pakistan (MT066231). They had close relationships with Asiatic, American, and European isolates.

The sequences of STV isolates were retrieved from GenBank^®^ (NCBI, USA) (Supplementary Table S3). The MSA of these isolates, based on complete genome sequences, indicated that STV isolates (OP548653, OP548652, and MT066231) had more than 98% sequence homology between them and other STV isolates with small numbers of mutations at different sites in the genome. Molecular phylogenetic analysis was performed and a tree was constructed on the bases of complete genome sequences using MEGA X, which revealed that STV isolates (OP548653, OP548652, and MT066231) had a close evolutionary relationship with Asiatic, European, and American isolates that infect *S. lycopersicum* and *Capsicum annuum* under greenhouse and field conditions. The highest similarity (100%) was observed between MN095716 and EF442780 isolates reported from Colombia and Mexico, respectively, and the lowest similarity (98.48%) was observed in OL471993, which is an isolate that originated from Slovenia. However, the average percentage homology of aligned sequences of all these clade isolates was >98% (Figure 4 and Table S4).

### 3.4. Comparison of Genetic Variability between STV Populations

**Figure 5:**
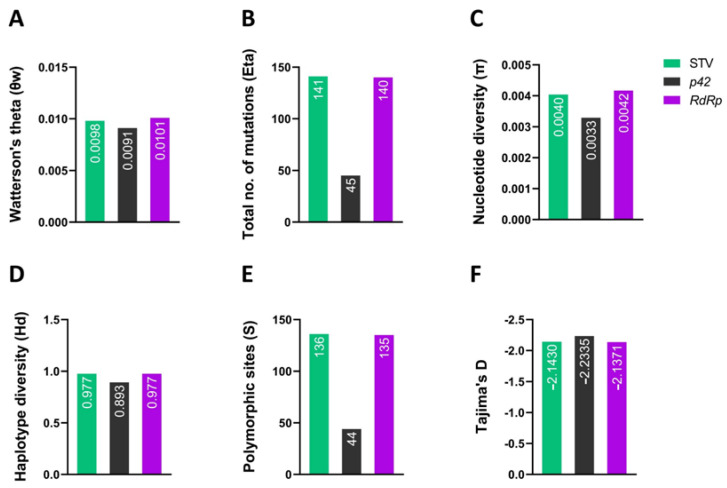
The parameters associated with genetic variability (diversity) were calculated for 44 STV isolates and their individual proteins (p42 and RdRp). These parameters were (**A**) Watterson’s theta (θw), (**B**) number of mutations (Eta), (**C**) nucleotide diversity (π), (**D**) haplotype diversity (Hd), (**E**) polymorphic sites (S), and (**F**) Tajima’s D.

Further, we analyzed the standing molecular diversity among 44 sequences of STV and compared the genetic variations between the p42 and RdRp genes. The genetic diversity of STV, determined at the whole genome level, revealed that 33 haplotypes were detected with the gene (haplotype) diversity (Hd) being 0.977. The number of segregating (polymorphic) sites for STV populations was 136, with 141 mutations (Eta). The standing nucleotide diversity (π) was estimated to be 0.00404. A statistically significant (*p* < 0.05) and highly negative value of Tajima’s D (−2.14304) among the STV sequences indicated the presence of excessive polymorphic sites (Figure 5 and Table 2). Similarly, a genetic diversity analysis was performed for p42 and RdRp coding sequences, which demonstrated that the values of the aforementioned parameters were higher for RdRp compared with p42, except for Tajima’s D value, which was significantly slightly more negative (−2.2335) for p42 sequences than for RdRP (−2.1371) (Figure 5F and Table 2). 

### 3.5. Possible Recombination Events Involved in the Genetic Diversity of STV

**Figure 6:**
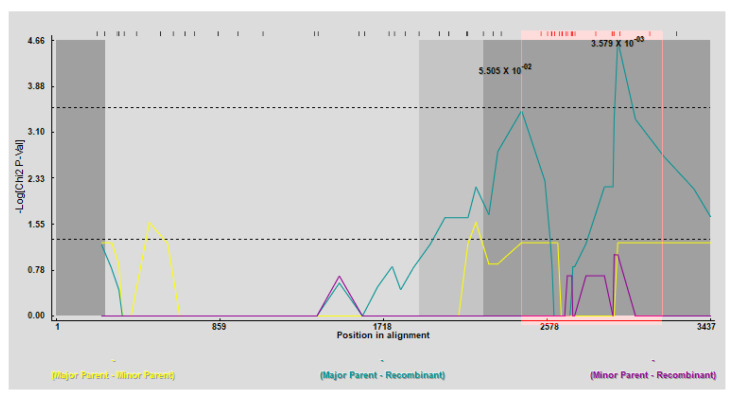
Recombination events detected in the full-length genome of STV populations. The *x*-axis indicates the position in alignment, while the *y*-axis denotes the Log[Chi2 P-Val]. Light green and light blue peaks indicate major and minor parents, respectively; the yellow peak marks both major and minor parents simultaneously. The recombination event corresponds to a recombinant isolate (OK309713) with major (OK309721) and minor (KT438549) parents.

To investigate the role of recombination in the standing genetic diversity existing among STV populations, we performed a recombination test using RDP, which revealed a total of three putative recombination events among 44 STV sequences. The first event was detected among 24 sequences with only one method (MaxChi, *p*-value 3.579 × 10^−3^). In this event, OK309713 (Turkish isolate) was designated as a recombinant sequence with a major parent OK309721 and an unknown minor parent (Figure 6 and Table 3). The second recombination event was found in 38 sequences with KY228384 (Chinese isolate) being recombinant with OK309710 and MF422617 as major and minor parents, respectively. The recombination signals were detected using two methods (Bootscan and 3Seq), with a significant *p*-value of 3.277 × 10^−2^ (Figure S1 and Table 3). Finally, the third recombination event was found only in three sequences and the recombinant sequence was KT438549 (Chinese isolate) with the major parent OK309708 and an unknown minor parent. This event was also supported using two methods (3Seq and SisterScan), with a significant *p*-value of 2.625 × 10^−2^ (Figure S2 and Table 3). Taken together, a lower-to-moderately significant impact of recombination was observed to be associated with existing genomic variation among the STV populations.

### 3.6. Analysis of Positive and Negative Selection

**Figure 7:**
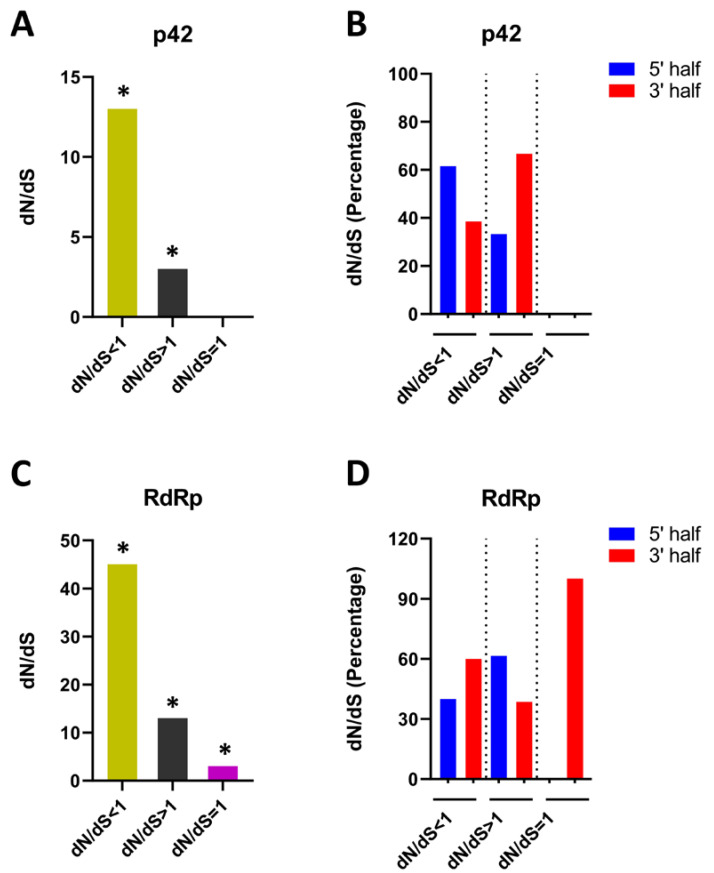
Estimation of positive (dN/dS > 1), negative (dN/dS < 1), and neutral (dN/dS = 1) selection acting upon codons of the major STV proteins (p42 and RdRp). (**A**) number and (**B**) percent distribution of p42 codons under positive, negative and neutral selection pressure, (**C**) number and (**D**) percent distribution of RdRp codons under positive, negative and neutral selection pressure. The * indicates that these sites were selected at the significance level of *p* < 0.1.

In order to gain a comprehensive understanding of the possible role of selection pressure in the evolution of STV, we analyzed the role of non-synonymous to synonymous substitutions (d_N_/d_S_) in shaping the genomic variations between STV populations. We compared the overall d_N_/d_S_ for the p42 and RdRp regions. The results based on the DataMonkey analysis showed that p42 was mainly evolving under negative or purifying selection pressure, as it contained a higher number (13) of codons with d_N_/d_S_ < 1 compared with only three sites under positive selection pressure (d_N_/d_S_ > 1). No codons within p42 sequences were detected to be evolving under neutral selection pressure (d_N_/d_S_ = 1 (Figure 7A). Further analysis revealed that the 5’ half of p42 exhibited more negatively selected sites (61.5%) compared with the 3’ half (38.5%) (Figure 7B). On the other hand, RdRp was observed to contain 45, 13, and 3 sites under negative, positive, and neutral selection pressure, respectively (Figure 7C). Interestingly, in contrast to p42, the distribution of negatively selected sites was lower (40%) in the 5′ half compared with the 3’ half (60%) of RdRp. Notably, all three positively selected sites were detected in the 3’ half of RdRp (Figure 7D). Results of the selection pressure analysis demonstrated that although negative selection pressure was the major factor acting upon p42 and RdRp, the distribution pattern of negatively and positively selected sites remained variable among both proteins.

## 4. Discussion

All kinds of cellular life forms are vulnerable to being parasitized by several diverse viruses, leading to multifaceted intra-host virus–virus interactions and evolution. STV is a persistent quarantine pathogen that spreads vertically through tomato seeds, infecting tomato plants without exhibiting obvious symptoms under a single infection condition, but in association with TYLCV, ToCV, TICV, TSWV, and ToMV, provokes ToYSD, exacerbating chlorotic, leaf yellowing, stunting, and fruit deformation symptoms (Figure 1B,C) [8,9,10,11,12,13]. In this study, for the first time, we conducted large-scale STV infection diagnosis and global evolutionary dynamics with its genetic characterization. An agile cryptic agent, namely, STV, in the aggression of ToYSD was identified in diseased greenhouse and field-grown tomatoes from various geographical localities with yellowing, stunting, and fruit size reduction symptomologies (Table S1). STV was detected in both single and mixed infections with the combination of different viruses across Beijing, China, and Punjab, Pakistan, with a variable rate of infections (Table 1). A high rate of STV infection was recorded under mixed-infection conditions with TYLCV, ToCV, TICV, TSWV, and ToMV in Beijing, China, and Punjab, Pakistan (Figure 2C,D). Remarkably, STV in a single infection did not produce obvious symptoms via regulating complex cellular pathways in tomato plants against pathogens through the capricious expression of endogenous plant miRNAs and vsiRNAs. However, in mixed infection, it most frequently interacts with other viruses, such as TYLCV, ToCV, CMV, and PepMV, and triggers ultra-structural modification in the host plant with severe disease induction and overt symptomologies [9,15,18,19,53]. Several studies revealed that multiple viral infections may lead to a great variety of multilayered intra-host virus–virus interactions involved in the virus recombination for evolution, suppression of host defense mechanisms, and synergism of viral pathogenicity [54,55,56,57]. The synergistically intricate interaction of TYLCV with ToCV and TICV drastically subverts the host defense mechanism and aggravates tomato leaf curl disease (TLCD) [54,58,59]. Sweet potato chlorotic stunt crinivirus (SPCSV) interacts with sweet potato feathery mottle potyvirus (SPFMV) and sweet potato mild mottle ipomovirus and triggers sweet potato viral disease (SPVD) and sweet potato severe mosaic disease (SPSMD) epidemics, respectively [55,60]. Similarly, mixed-infection African cassava mosaic begomovirus (ACMV), cassava mosaic Madagascar begomovirus (CMMGV), East African cassava mosaic begomovirus (EACMV), East African cassava mosaic Kenya begomovirus (EACMKV), East African cassava mosaic Malawi begomovirus (EACMMV), East African cassava mosaic Zanzibar begomovirus (EACMZV), and South African cassava mosaic begomovirus (SACMV) resulted in a global cassava mosaic disease (CMD) pandemic [61,62].

The genomic organization of STV indicates that it consists of a single dsRNA molecule with two partially overlapping ORFs, encoding p42 (CP) from ORF1 and RdRp from ORF2 through +1 ribosomal frameshifting as a fused product. The presence of putative slippery sites followed by a pseudoknot configuration in the STV genome was considered to be significantly involved in +1 ribosomal frameshifting [8]. STV is an exclusive virus, its genomic characteristics are the amalgam of two families, namely, *Partitiviridae* and *Totiviridiae*. The family *Partitiviridae* includes dsRNA viruses, which infect fungi and plants and possess a divided dsRNA genome comprised of dsRNA1 (encoding RdRp) and dsRNA2 (encoding viral CP). However, the family *Totiviridiae* contains dsRNA viruses that infect protozoal and fungal hosts and have an undivided dsRNA genome with two partially overlapping ORFs that encode viral CP and RdRp expressed via ribosomal frameshifting [8,63,64,65]. For genomic characterization and phylogenetic analysis, full-length STV genomes were constructed from high-disease-incidence regions (Fangshan, Tongzhou, and Faisalabad) through yeast homologous recombination cloning and submitted to NCBI GenBank (OP548653, OP548652, and MT066231) to expand the virus genomic data and to support the scientific community in further biological annotation and evolutionary dynamics. 

To acquire a better understanding of evolutionary genomics and to attain deeper insights into the evolution rate of STV, we analyzed full-length genome sequences of 44 STV isolates by using wide-ranging computational tools and inferred the molecular evolutionary genomics. Molecular phylogenetic analysis and sequence alignments indicated that all STV isolates, including those from Asia, Europe, and America, had close (>98%) phylogenetic relationships with lower genetic variability (Figure 4). Moreover, the analysis of genetic variability showed that the genetic variability for RdRp was higher compared with p42 (Figure 5). The apparently higher genetic variability observed for the RdRp region might be correlated with its larger coding sequence compared with that of p42. Further, it would be interesting to investigate how ORFs correlate and/or govern the genetic diversity among STV populations. The number of recent studies on molecular evolutionary analysis has been increasing, including those on phylodynamics and temporal evolutionary characteristics of various plant viruses based on one or few gene sequences, including the VPg gene of potato potyvirus Y (PVY) [66]; the NABP and CP genes of potato potyvirus M (PVM) [67]; and the P3, CI, and Nib genes of PVY [68]. To mitigate any ambiguity in extrapolating the evolutionary dynamics of a virus based on a single or a few genes to an entire virus species, NGS-based sequencing followed by de novo assembly would provide nearly complete genomic sequences, which could be employed to actually develop a far more standardized portrayal of the genetic diversity of virus populations [69,70]. 

Evolution and genetic diversity are considered to be driven by recombination events [71,72,73,74,75]. No study has been reported regarding the genetic diversity of STV that combines its global population. In our results, a total of three recombination events were detected among 44 STV sequences (population). The first recombination event was found among 24 sequences with a Turkish isolate (OK309713), which was recombinant with OK309721 as a major parent and an unknown minor parent (Figure 6 and Table 3). The second event was detected among 38 sequences with a Chinese isolate (KY228384), which was designated as a recombinant sequence with OK309710 as the major parent and MF422617 as the minor parent (Figure S1 and Table 3). Finally, the third event was detected only in three sequences with the recombinant sequence, namely, a Chinese isolate (KT438549), which was recombinant with OK309708 as a major parent and an unknown minor parent (Figure S2 and Table 3). Thus, recombinant events in RNA viruses are most common due to assortments of the RNA-segmented viral genomes [76,77,78]. Both intraspecific homologous and interspecific non-homologous recombination are considered to be the most frequent and significant in the evolution of poleroviruses, such as sugarcane yellow leaf polerovirus (ScYLV) [79,80], cotton leaf dwarf polerovirus (CLRDV) [81], brassica yellows polerovirus (BrYV) [82], faba bean polerovirus 1 (FBPV-1) [83], and cucurbit aphid-borne yellows polerovirus (CABYV) [76,84]. Several factors, such as increased viral replication, mixed infections, expanded host range, and vector, are known to greatly regulate recombination [85]. Recombination could play a substantial role in the evolution of STV populations, as most genes involved in certain compatible interactions may be evolving under selection pressure from their hosts and have a tendency to accumulate variations faster than other parts of the genome. Notably, gene mutations or recombination can influence the biological functions regulated by viral proteins. However, how recombination affects the biological functions related to these genes is a matter of consideration that should be investigated more in the future.

Furthermore, our findings showed that STV populations are mostly evolving under negative/purifying selection pressure. To gain an in-depth understanding of this selection factor at the gene level, we opted to estimate d_N_/d_S_ for the p42 and RdRp genes. Our results demonstrated that the majority of codons remained under negative selection for each gene, with an average d_N_/d_S_ ratio of <1 (Figure 7), indicating that while negative selection pressure was the main factor acting upon p42 and RdRp, the distribution pattern of negatively and positively selected sites remained variable among both proteins. This is in accordance with previous studies, which concluded that genes of turnip yellows polerovirus (TuYV) and cotton leaf curl Multan begomovirus (CLCuMuV) evolved under negative selection pressure [86,87,88]. 

The present study illustrated how the analysis of genetic diversity and the structure of plant virus populations is essential for understanding the evolutionary biology of plant viruses related to the dynamics of virus populations and associated disease epidemiology. Meanwhile, evolutionary forces (mutation, recombination, and persistent selection pressure), virus–host interactions, and enhanced host immunity may favor rapid virus evolution and reshape its pathogenicity and disease epidemiology. However, these evolutionary dynamics affecting virus pathogenicity and disease epidemiology are worth mentioning in modern plant virology.

## 5. Conclusions

The present study demonstrates the infection status and evolutionary dynamics of STV, which is the most prevalent in all tomato production regions of the world, affecting sustainable food production in association with other plant viruses. This virus was transmitted vertically and widely distributed, showing high disease incidence in major tomato production fields in Beijing, China, and Punjab, Pakistan. STV had a close evolutionary relationship with its other isolates, but the genetic variability observed for RdRp was higher than that of p42. Consequently, it evolved under a strong purifying selection process. These findings provide solid foundations for the development and implementation of novel approaches for the timely diagnosis and long-term management of STV.

## Figures and Tables

**Table 1:** STV infection dynamics under field conditions and confirmation through laboratory analysis and high-throughput sequencing technology (HTS).

Sample Locality		GPS (Latitude, Longitude)	Type of Sample (Production)	Rate of ToYSD Infection (%) ^1^		Rate of STV Infection (%)		Multiple-Virus Infections		
				ToYSD Prevalence ^a^	ToYSD Incidence ^b^	RT-PCR	Sanger	RT-PCR	Sanger	HTS
Beijing, China	Fangshan	39°44′4.67″ N, 116°11’30.7″ E	Leaf (greenhouse)	80.00	75.00	58.33	√	STV, TYLCV, ToCV, TSWV, ToMV	√	√
	Changping	40°10′39.36″ N, 116°23′59.17″ E	Leaf (greenhouse)	75.00	71.42	57.14	√	STV, TYLCV, ToCV, TSWV	√	//
	Tongzhou	39°54′35.87″ N, 116°39′23.17″ E	Leaf/seed (greenhouse)	83.33	76.66	53.33	√	STV, TSWV, TYLCV, ToCV, ToMV, TICV	√	√
	Shunyi	40°7′49.25″ N, 116°39′16.74″ E	Leaf (greenhouse/open field)	66.66	62.50	37.50	√	STV, TYLCV	√	//
	Miyun	40°22′36.95″ N, 116°50′35.04″ E	Leaf (greenhouse)	60.00	58.33	33.33	√	STV, TYLCV, ToCV	√	//
	Pinggu	40°7′34.61″ N, 117°15′31.86″ E	Leaf (greenhouse)	60.00	57.14	35.71	√	STV, TSWV, TYLCV, ToCV	√	//
	Daxing	39°47′29.3″ N, 116°29′48.44″ E	Leaf (greenhouse)	75.00	66.66	41.66	√	TYLCV, STV, TICV, ToMV	√	//
	Yanqing	40°31′4.33″ N, 115°54′47.89″ E	Leaf (greenhouse)	66.66	57.14	28.57	√	STV, TYLCV, ToCV	√	//
	Overall STV incidence					43.19				
Punjab, Pakistan	Faisalabad	31°27′1.32″ N, 73°8′5.86″ E	Leaf/seed (Greenhouse/open field)	87.50	83.33	50.00	√	STV, ToCV, TYLCV, ToMV	√	√
	Multan	30°9′26.85″ N, 71°31′29.69″ E	Leaf/seed (open field)	75.00	62.50	37.50	√	TYLCV, STV, ToCV	√	//
	Bahawalpur	29°21′15.66″ N, 71°41′27.84″ E	Leaf/seed (open field)	60.00	50.00	30.00	√	STV, TYLCV, ToCV	√	//
	Lahore	31°31′13.33″ N, 74°21′31.49″ E	Leaf/seed (open field)	80.00	71.42	42.85	√	STV, TYLCV, ToCV	√	//
	Overall STV incidence					40.08				

Abbreviations: GPS, global positioning system; STV, southern tomato amalgavirus; ToCV, tomato chlorosis crinivirus; TICV, tomato infectious chlorosis crinivirus; ToMV, tomato mosaic tobamovirus, TSWV, tomato spotted wilt orthotospovirus; TYLCV, tomato yellow leaf curl begomovirus; ToYSD, tomato yellow stunt disease. The symbol “√” denotes confirmed viral infection through sequencing (Sanger/HTS), while “//” indicates that NGS was not applied. **^1^** The rate of ToYSD infection (prevalence and incidence) was calculated using the following equations: ^a^ $\mathrm{ToYSD}\;\mathrm{prevalence}=\frac{X}{Y}\times 100$, where X is the number of sample production localities (greenhouses/fields) with visible ToYSD symptoms and Y is the total number of sample production localities observed in a region [26]. ^b^$\;\mathrm{ToYSD}\;\mathrm{incidence}=\frac{\left(N-n\right)}{N}\times 100$, where N is the total number of samples under observation and n is the total number of healthy samples without ToYSD symptoms and viral infections [26].

**Table 2:** Estimation of molecular genetic diversity among full genomes and individual genes (p42 and RdRp) of STV isolates.

Dataset.	No. of Sequences	No. of Analyzed Sites	S	H	Hd	π	θw		Eta	Neutrality Test
							Per Site	Per Sequence		Tajima’s D
STV	44	3305	136	33	0.977	0.00404	0.00946	31.264	141	−2.14304 *
p42	44	1134	44	23	0.893	0.00329	0.00892	10.115	45	−2.23347 **
RdRp	44	3190	135	33	0.977	0.00417	0.00973	31.034	140	−2.13714 *

S, number of segregating (polymorphic) sites; H, number of haplotypes; Eta, the total number of mutations; Hd, haplotype diversity; θw, Watterson’s theta; π, nucleotide diversity. *, statistically significant (*p* ≤ 0.05); **, statistically significant (*p* ≤ 0.01).

**Table 3:** Description of recombination events detected using RDP in the full-length genome and RdRp region of globally reported STV populations.

Recombination Event	Sequences Detected with Recomb. Event	Recombinant Sequence		Recombination Breakpoints without (with) Gaps		Parental Sequences		Detection Methods ^1^	*p*-Value ^2^
		Isolate	Country	Begin	End	Major	Minor		
STV									
1	24	OK309713	Turkey	2347 (2444)	3089 (3186)	OK309721	KT438549	M	3.579 × 10^−03^
2	38	KY228384	China	1078 (1082)	1284 (1288)	OK309710	MF522617	B3	3.227 × 10^−02^
3	3	KT438549	China	2440 (2444)	3182 (3186)	OK309708	OK309721	S3	2.625 × 10^−02^
RdRp									
1	26	OK309713	Turkey	2308 (2308)	2994 (2994)	OK309721	Unknown	M	1.758 × 10^−02^
2	2	OK309710	Turkey	945 (945)	1188 (1188)	KY228384	Unknown	3	3.277 × 10^−02^

^1^ R, RDP; G, GeneConv; B, Bootscan; M, MaxChi; C, CHIMAERA; S, SisScan; 3, 3SEQ. ^2^ The described *p*-value corresponds to the calculated *p*-value for the event in question, which was detected using the program in bold and underlined.

## Data Availability

All sequence data can be obtained from https://www.ncbi.nlm.nih.gov/genbank (accessed on 27 April 2022) using the Genbank accession numbers mentioned in Supplementary Table S3.

## Supplementary Materials

The following supporting information can be downloaded from https://www.mdpi.com/article/10.3390/v14112481/s1. Figure S1. The second recombination event detected among 38 full-length genomic sequences of STV; Figure S2. The third recombination event detected in three full-length sequences of STV; Table S1. Tomato samples collected from different regions of China and Pakistan were employed in this study for the detection of STV and confirmation of its infection dynamics under single- and mixed-infection conditions with variable rates of infection; Table S2. Primer sequences used in this study for the detection of different plant viruses and amplification, as well as molecular cloning of STV; Table S3. STV reference isolates used in this study with a detailed description, including the accession number, name of isolate, sequence length, country, and host; Table S4. The average percentage sequence homology of aligned sequences of STV populations.
