# Supplementary material for: Molecular Detection of Southern Tomato Amalgavirus Prevalent in Tomatoes and Its Genomic Characterization with Global Evolutionary Dynamics
Source: Viruses. 2022 Nov 9;14(11):2481. doi: 10.3390/v14112481 (PMC9693158; doi:10.3390/v14112481)
Supplement: Supplementary file 1 [file viruses-14-02481-s001.zip › Supplementary Figures and Tables.pdf]

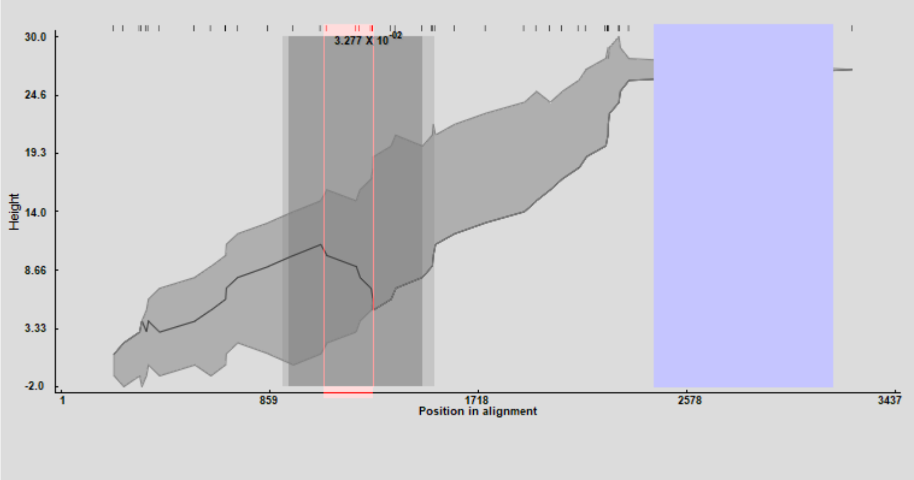

Figure S1. The second recombination event detected among 38 full-length genomic sequences of STV.

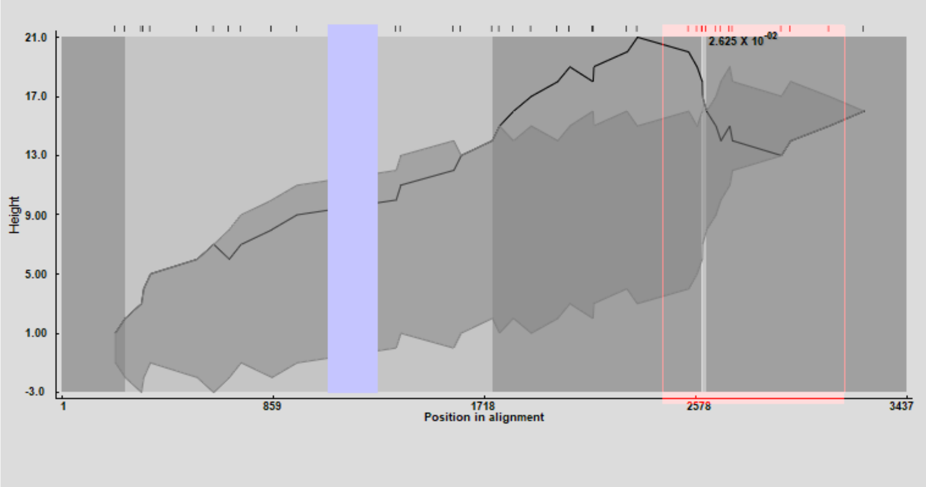

Figure S2. The third recombination event detected in 03 full-length sequences of STV.

Table S1. Tomato samples collected from different regions of China and Pakistan were employed in this study for the detection of STV and confirmation of its infection dynamics under single and mixed infection conditions with its variable rates of infections.

Sample locality		GPS (Latitude, Longitude)	Sample designation	Type of sample (Production)	Symptoms	Suspected viral infections	Rate of ToYSD infection (%) [1]		Detection and confirmation of STV		Confirmed rate of STV infection (%)[1]	Percentage of STV single infection	Percentage of STV mixed infection in different combinations of viruses			
							ToYSD Incidence [a]	ToYSD Prevalence [b]	RT-PCR	Sanger	STV Incidence	STV	STV+TYLCV	STV+TYLCV+ToCV	STV+TYLCV+ToCV+TSWV	STV+TYLCV+ToCV+TSWV+ToMV
Beijing, China	Fangshan	39°44'4.67"N, 116°11'30.7"E	FS-08122017-1	Leaf (Greenhouse 1)	Typical yellowing, leaf curling, mild crinkling, and deformations	TYLCV, STV, ToCV	75.00	80.00	STV, TYLCV	STV, TYLCV	58.33	25.00	16.66	08.33	00.00	08.33
			FS-08122017-2		Leaf yellowing, chlorosis, mild crinkling, and	TYLCV, STV, ToCV, TICV			TYLCV, STV, ToCV	TYLCV, STV, ToCV						

				fruit deformations											
		FS-08122017-3		Typical yellowing, leaf curling, mottling, and deformations	TYLCV, STV, ToCV, TSWV			STV	STV						
		FS-08122017-4		Leaf chlorosis, mosaic, mottling, and yellowing without crinkling	TYLCV, STV, ToCV, CMV, ToMV, PVY			-	-						
		FS-08122017-5	Leaf (Greenhouse 2)	Leaf crinkling, mosaic, and dark green with mites	ToMV, CMV, PVY			-	-						
		FS-08122017-6		Leaf yellowing, crinkling, and deformations	STV, ToMV, TYLCV, PVY			STV	STV						
		FS-08122017-7		Typical leaf curling and mild crinkling	TYLCV, ToCV, TICV			TY-LCV	TY-LCV						
		FS-08122017-8	Leaf (Greenhouse 3)	Typical yellowing, necrotic patches, and dark green leaves	TYLCV, STV, ToCV, TSWV, CMV, ToMV, TICV			STV, TSWV, TY-LCV, ToCV, ToMV	STV, TSWV, TY-LCV, ToCV, ToMV, TICV*						
		FS-08122017-9		Leaf yellowing, chlorosis, mild crinkling, and fruit deformations	TYLCV, STV, ToCV,			STV, TY-LCV	STV, TY-LCV						
		FS-08122017-10		Leaf crinkling and deformation with whiteflies infestation	TYLCV, ToCV			-	-						
		FS-08122017-11	Leaf (Greenhouse 4)	Leaf curling with mites and whiteflies	STV, TYLCV, ToCV			TY-LCV	TY-LCV						
		FS-08122017-12		Leaf chlorosis, mosaic, mottling, and yellowing	TYLCV, STV, ToCV, CMV, ToMV, PVY			STV	STV						
		XTS-19012018-1		Leaf yellowing, chlorosis, mosaic, mottling, and fruit reduction	TYLCV, STV, ToCV, CMV, ToMV, PVY			STV, TY-LCV, ToCV	STV, TY-LCV, ToCV						
Changping	40°10'39.36"N, 116°23'59.17"E	XTS-19012018-2	Leaf (Greenhouse1)	Leaf yellowing, chlorosis, mosaic, mottling, and fruit reduction	TYLCV, STV, ToCV, CMV, ToMV,	71.42	75.00	TY-LCV, STV	TY-LCV, STV	57.14	14.28	14.28	7.14	7.14	00.00
		XTS-19012018-3		Leaf chlorosis, mosaic, mott-	TYLCV, STV, ToCV,			STV, TY-LCV,	STV, TY-LCV,						

				ling, yellowing, and fruit deformation	ToMV, PVY			ToCV ToMV	ToCV, ToMV						
		XTS-19012018-4		Leaf crinkling and deformations	TYLCV, ToCV, TSWV			-	-						
		XTS-19012018-5		Typical yellowing, necrotic patches, and dark green leaves	TYLCV, STV, ToCV, TSWV, CMV			STV, ToCV, TSWV, TY-LCV	STV, TSWV, TY-LCV, ToCV						
		XTS-31102018-1	Leaf (West greenhouse)	Typical leaf yellowing and leaf curling with mild crinkling	TYLCV, STV, ToCV			STV	STV						
		XTS-31102018-2		Typical leaf curling with mild crinkling	TYLCV, ToCV			-	-						
		XTS-31102018-3		Leaf chlorosis, mosaic, mottling, and yellowing	TYLCV, STV, ToCV, CMV, ToMV, PVY			STV, ToCV	STV, ToCV						
		XTS-31102018-4		Leaf chlorosis, mosaic, mottling, and yellowing	TYLCV, STV, ToCV, CMV, ToMV, PVY			TY-LCV	TY-LCV						
		XTS-31102018-5		Crinkle, mosaic, and dark green patches on leaves	ToMV, CMV, PYV			-	-						
		XTS-31102018-6	Leaf (East greenhouse)	Emerging leaves becoming yellow and mild crinkle	TYLCV, STV, ToMV, CMV			TY-LCV, STV	TY-LCV, STV						
		XTS-31102018-7		Emerging leaves becoming yellow and mild crinkle	TYLCV, STV, ToMV, CMV			-	-						
		XTS-31102018-8		Leaves become smaller and darker, and infested with mites	STV, ToCV, CMV			STV	STV						
		XTS-31102018-9		Leaves become smaller and darker, and infested with whiteflies	ToCV, CMV, TYLCV			-	-						
Tongzhou	39°54'35.87"N, 116°39'23.17"E	TZ-12042018-1	Leaf (Greenhouse 1)	Leaf chlorosis, mosaic, mottling, and yellowing	TYLCV, STV, ToCV, CMV, ToMV, PVY	76.66	83.33	TY-LCV, STV	TY-LCV, STV	53.33	30.00	10.00	10.00	00.00	03.33
		TZ-12042018-2		Leaf chlorosis, mosaic, and mottling	TYLCV, ToCV, CMV,			-	-						

			ToMV, PVY		
TZ-12042018-3		Typical yellowing, necrotic patches, and dark green leaves	TYLCV, STV, ToCV, TSWV, CMV, ToMV, TICV	STV, ToCV, TSWV, TYLCV, ToMV	STV, TSWV, TYLCV, ToCV, ToMV, TICV*
TZ-12042018-4		Leaf yellowing, deformed, and reduce fruit size	TYLCV, STV, ToCV	STV	STV
TZ-12042018-5	Leaf (Greenhouse 2)	Leaf chlorosis, yellowing, mosaic, and mottling	TYLCV, STV, ToCV	STV, TYLCV, ToCV	STV, TYLCV, ToCV
TZ-12042018-6		No obvious symptoms look healthy sample	STV	STV	STV
TZ-12042018-7		Stunted plant growth having alternate patches on leaves	STV, ToMV, CMV	STV	STV
TZ-12042018-1		Leaf yellowing, crinkling, deformation	STV, ToMV, ToCV, TYLCV, TICV	STV, ToCV, TYLCV	STV, ToCV, TYLCV
TZ-12042018-2		Leaf chlorosis, mosaic, and mottling	TYLCV, ToCV, CMV, ToMV, PVY	-	-
TZ-12042018-3	Leaf (Greenhouse 3)	Leaf chlorosis and deformation	TYLCV, STV, ToCV, CMV, ToMV	STV	STV
TZ-12042018-4		Leaf yellowing, chlorosis, mild crinkling, and fruit deformations	TYLCV, STV, ToCV, TICV	STV, TYLCV, ToCV	STV, TYLCV, ToCV
TZ-12042018-5		Mild crinkling, and fruit deformations	TYLCV, ToCV, ToMV, CMV	-	-
TZ-18122019-1			TYLCV	-	-
TZ-18122019-2	Seed, F1 generation (Seed source 1)	Directly obtained from the seed source	TYLCV, STV	STV, TYLCV	STV, TYLCV
TZ-18122019-3			STV, TYLCV	STV	STV
TZ-18122019-4			TYLCV	TYLCV	TYLCV

		TZ-18122019-5			STV, TYLCV			STV	STV						
		TZ-18122019-6			STV, TYLCV			-	-						
		TZ-18122019-1	Seed, F1 generation (Seed source 2)	Directly obtained from the seed source	TYLCV, STV			-	-						
		TZ-18122019-2			TYLCV, STV			STV	STV						
		TZ-18122019-3			TYLCV, STV			STV	STV						
		TZ-18122019-4			STV, TYLCV			-	-						
		TZ-18122019-5			STV, TYLCV			STV	NA						
		TZ-18122019-6			TYLCV			-	-						
		TZ-18122019-1	Seed, F1 generation (Seed source 3)	Directly obtained from the seed source	TYLCV, STV			STV	STV						
		TZ-18122019-2			TYLCV, STV			-	-						
		TZ-18122019-3			TYLCV, STV			TYLCV	TYLCV						
		TZ-18122019-4			STV, TYLCV			STV, TYLCV	STV, TYLCV						
		TZ-18122019-5			STV, TYLCV			TYLCV	TYLCV						
		TZ-18122019-6			TYLCV			-	-						
Shunyi	40°7'49.25"N, 116°39'16.74"E	SY-15062018-1	Leaf (Greenhouse 1)	Leaf yellowing and necrotic spot on fruit	STV, TSWV, TYLCV	62.50	66.66	STV	STV	37.50	25.00	12.50	00.00	00.00	00.00
		SY-15062018-2		Leaf chlorosis and deformation	STV, ToCV, TYLCV			-	-						
		SY-15062018-3		Leaf yellowing, chlorosis, and fruit malformation	STV, TSWV, TYLCV, ToCV			STV, TYLCV	STV, TYLCV						
		SY-15062018-4		Mild mosaic symptoms	ToMV TYLCV, CMV			-	-						
		SY-15062018-5	Leaf (Open field 1)	Mild crinkling, and fruit deformations	TYLCV, ToCV, ToMV, CMV, TICV			-	-						
		SY-15062018-6		Mild yellowing and crinkling	STV, ToMV TYLCV, CMV			STV	STV						

		SY-15062018-7		Leaf curling infested with whiteflies	STV, ToCV TYLCV			-	-						
		SY-15062018-8		Healthy sample	Control			-	-						
		MY-15062018-1		Typical yellowing, chlorosis between veins, and having no crinkle	TYLCV, STV, ToCV, CMV			TYLCV, STV	TYLCV, STV						
		MY-15062018-2	Leaf (Greenhouse 1)	Typical yellowing, chlorosis between veins, and having no crinkle	TYLCV, STV, ToCV, CMV			TYLCV, STV, ToCV	TYLCV, STV, ToCV						
		MY-15062018-3		Mild mosaic symptoms	ToMV, CMV, PVY			-	-						
		MY-15062018-4		No obvious symptoms look healthy sample	Control			-	-						
		MY-15062018-1		Mosaic, green island, and mild crinkle	ToCV CMV, PVY			-	-						
Miyun	40°22'36.95"N, 116°50'35.04"E	MY-15062018-2		Leaf chlorosis between veins and having no crinkle	TYLCV, STV, ToCV, CMV	58.33	60.00	TYLCV	TYLCV	33.33	08.33	16.66	08.33	00.00	00.00
		MY-15062018-3		Leaf yellowing, chlorosis, and having no crinkle	TYLCV, STV, ToCV, CMV			STV	STV						
		MY-15062018-4	Leaf (Greenhouse 2/3)	Leaf yellowing, chlorosis, and having no crinkle	TYLCV, STV, ToCV, CMV			-	-						
		MY-15062018-5		Leaf yellowing, chlorosis, and having no crinkle	TYLCV, STV, ToCV, CMV			TYLCV, STV	TYLCV, STV						
		MY-15062018-6		Leaf curling and crinkling	ToMV, ToCV, TYLCV			TYLCV	TYLCV						
		MY-15062018-7		Leaf yellowing, crinkling, and deformation	STV, ToMV, ToCV, TYLCV			-	-						
		MY-15062018-8		Leaf crinkling and deformation	ToMV, ToCV, TYLCV			TYLCV	TYLCV						
		PG-08112018-1		Leaf crinkling and deformation with whiteflies infestation	TYLCV, ToCV, TICV, TSWV			-	-						
Pinggu	40°7'34.61"N, 117°15'31.86"E	PG-08112018-2	Leaf (Greenhouse 1)	Leaf yellowing and fruit deformation	STV, TSWV	57.14	60.00	STV	STV	35.71	14.28	14.28		07.14	00.00
		PG-08112018-3		Leaf yellowing and fruit deformation	STV, TSWV			-	-						

		PG-08112018-4		Emerging leaves becoming yellow and mild crinkle	TYLCV, STV, ToMV, CMV			STV, TY-LCV	STV, TY-LCV						
		PG-08112018-5		No obvious symptoms look healthy sample	Control			-	-						
		PG-08112018-6		Leaf yellowing and stunting	STV, TYLCV, ToCV, ToMV			-	-						
		PG-08112018-7	Leaf (Greenhouse 2)	Leaf yellowing, curling, crinkling, and stunting	STV, TYLCV, ToCV, ToMV, TICV			STV, TY-LCV	STV, TY-LCV						
		PG-08112018-8		Leaf yellowing, curling, and stunting	STV, TYLCV, ToCV, ToMV			-	-						
		PG-08112018-1		Leaf curling and crinkling	TYLCV, ToMV			-	-						
		PG-08112018-2		Leaf yellowing and crinkling	STV, TYLCV, ToMV			STV	STV						
		PG-08112018-3		Leaf curling and crinkling	TYLCV, ToMV, PVY			-	-						
		PG-08112018-4	Leaf (Greenhouse 3)	Typical yellowing, necrotic patches, and dark green leaves	TYLCV, STV, ToCV, TSWV, CMV			STV, ToCV, TSWV, TY-LCV	STV, TSWV, TY-LCV, ToCV						
		PG-08112018-5		No obvious symptoms look healthy sample	STV			-	-						
		PG-08112018-6		Mosaic, green island, and mild crinkle	TYLCV, ToCV, TSWV, CMV			-	-						
		DX-12042019-1	Leaf (Greenhouse 1)	Leaf chlorosis, mosaic, mottling, and yellowing without crinkling	TYLCV, STV, ToCV, CMV, ToMV, PVY			TY-LCV, STV, ToCV, ToMV	TY-LCV, STV, ToMV						
Daxing	39°47'29.3"N, 116°29'48.44"E	DX-12042019-2		Mosaic, mottling, and yellowing without crinkling	TYLCV, STV, ToCV, CMV, ToMV, PVY	66.66	75.00	STV, TY-LCV	STV, TY-LCV	41.66	16.66	08.33	08.33	00.00	00.00
		DX-12042019-3		Mosaic, mottling, and stunting	TYLCV, ToCV, CMV, ToMV			-	-						
		DX-12042019-4		Mosaic, mottling, and stunting	TYLCV, STV, ToCV, CMV, ToMV			-	-						

		DX-12042019-5		Leaf yellowing and curling	TYLCV, STV, ToCV, CMV, ToMV			STV	STV						
		DX-12042019-6		Typical yellowing, leaf curling, mild crinkling, and deformations	TYLCV, STV, ToMV, ToCV, CMV			TY-LCV	TY-LCV						
		DX-12042019-7	Leaf (Greenhouse 2)	Typical yellowing, leaf curling, mild crinkling, and deformations	TYLCV, STV, ToMV, CMV, ToCV			TY-LCV, STV, ToCV	TY-LCV, STV, ToCV						
		DX-12042019-8		Mild mosaic symptoms	ToMV, PVY, CMV			-	-						
		DX-12042019-1		Leaf mottling and deformation and ring necrosis on fruits	TSWV, ToCV, TYLCV, PVY, TICV			TSWV	TSWV						
		DX-12042019-2		Mild leaf mottling and necrotic ring spot on fruits	TSWV, STV, ToCV, TYLCV, PVY			-	-						
		DX-12042019-3	Leaf (Greenhouse 3)	Leaf yellowing, mosaic mottling, and necrosis	STV, TYLCV, ToCV, ToMV, CMV, PVY			STV	STV						
		DX-12042019-4		Leaf mottling, fruit mottling, and uneven ripening	TSWV, TYLCV, ToCV, ToMV, CMV, PVY			-	-						
		YQ-27062019-1		Typical yellowing and leaf curling	TYLCV, STV, ToCV			TY-LCV, STV	TY-LCV, STV						
		YQ-27062019-2	Leaf (Greenhouse 1)	Typical yellowing and leaf curling	TYLCV, STV, ToCV			TY-LCV, STV, ToCV	TY-LCV, STV, ToCV						
Yanqing	40°31'4.33"N, 115°54'47.89"E	YQ-27062019-3		Yellowing, chlorosis between veins, no crinkling	TYLCV, STV, ToCV, CMV ToMV, PVY	57.14	66.66	TY-LCV	TY-LCV	28.57	00.00	14.28	14.28	00.00	00.00
		YQ-27062019-4		Mosaic, green island, and mild crinkle	ToMV, CMV, PVY			-	-						
		YQ-27062019-5	Leaf (Greenhouse 2)	Mosaic, green island, and mild crinkle	STV, TYLCV, ToMV, CMV, PVY			TY-LCV	TY-LCV						
		YQ-27062019-6		Mosaic, green island, and mild crinkle	TYLCV, ToMV,			TY-LCV	TY-LCV						

						CMV, PVY										
			YQ-27062019-7	Leaf (Greenhouse 3)	No symptoms (health sample)	TYLCV, STV, ToCV, CMV ToMV, PVY			-	-						
<u>Beijing, China</u><br>[Total harvested samples= 109, Negative= 45 samples, STV infection= 21 samples, TYLCV infection= 13 samples, TSWV infection= 1 sample, STV+TYLCV infection= 14 samples, STV+ToCV infection= 1 sample, STV+TYLCV+ToCV infection= 8 samples, STV+TYLCV+ToCV+TSWV infection= 2 samples, STV+TYLCV+ToCV+TSWV+ToMV infection= 2 samples, STV+TYLCV+ToCV+ToMV infection= 1 sample, TYLCV+ STV+ ToMV infection= 1 sample]<br>*Samples, FS-08122017-8 and TZ-12042018-3, were subjected to the Next generation sequencing technology and STV was detected with multiple viruses (STV, TSWV, TYLCV, ToCV, ToMV, and TICV).<br><u>Rate of infections</u><br>Negative= 41.28 %<br>STV single infection= 19.26 %<br>STV mixed infection= 26.60%<br>STV+TSWV+TYLCV+ToCV+ToMV+TICV= 01.83%												19.26	12.84	07.33	01.83	01.83
Punjab, Pakistan	Faisalabad	31°27'1.32"N, 73°8'5.86"E	FSD-13022019-Nagina	Seed (ARRI, Greenhouse 1)	Pronounce chlorotic, yellowing, and leaf curling	TYLCV, STV	83.33	87.50	STV, TYLCV	STV, TYLCV	50.00	08.33	25.00	08.33	00.00	00.00
			FSD-13022019-Nagina		Pronounce chlorotic, yellowing and leaf curling	STV, TYLCV			TYLCV	TYLCV						
			FSD-13022019-Rio Grande	Seed/ Leaf (ARRI, Greenhouse 2)	Emerging leaves becoming yellow, mild mosaic and crinkle	STV, TYLCV, ToCV, ToMV, CMV			STV, ToCV, TYLCV, ToMV	STV, ToCV, TYLCV, ToMV *						
			FSD-13022019-Thorgal	Seed/ Leaf (ARRI, East greenhouse)	Leaves are becoming smaller and darker infested with whiteflies and mites.	TYLCV, ToCV, ToMV, CMV			-	-						
			FSD-13022019-Morgal		Typical chlorosis, yellowing and leaf curling	STV, TYLCV, ToCV, ToMV, CMV, PVY			TYLCV	TYLCV						
			FSD-13022019-SBS-292	Seed (PPRI, South greenhouse)	Typical chlorosis, yellowing and leaf curling	STV, TYLCV,			STV, TYLCV	STV, TYLCV						
			FSD-13022019-GSL-198	Seed/ Leaf (PPRI, South greenhouse)	Leaf yellowing, mild mosaic mottling	STV, TYLCV, ToCV, ToMV, CMV, PVY			STV, TYLCV, ToCV	STV, TYLCV, ToCV						
			FSD-13022019		Leaf yellowing, mild mosaic mottling	STV, TYLCV, ToCV,			STV, TYLCV	STV, TYLCV						

		-Baby Red			ToMV, CMV, PVY										
		FSD-13022019-Nemador		Typical leaf curling, and deformation	TYLCV			-	-						
		FSD-13022019-VRI-575	Seed/ Leaf (VRI, Glass-house)	Stunting, chlorotic, and mosaic mottling	STV, TYLCV, ToCV, ToMV, CMV, PVY			STV	STV						
		FSD-13022019-Naqeeb-1		Mosaic and mottling	TYLCV, ToMV, CMV, PVY			-	-						
		FSD-13022019-Naqeeb-2	Seed (VRI, Glass-house)	No obvious symptoms look healthy sample	STV, TYLCV			-	-						
Multan	30°9'26.85"N, 71°31'29.69"E	MTN-16052020-Money Maker-1	Seed/ Leaf (Open field 1)	Typical yellowing, leaf curling, mild crinkling, and deformations	TYLCV, STV, ToMV, CMV, ToCV	62.50	75.00	TYLCV, STV, ToCV	TYLCV, STV, ToCV	37.50	12.50	12.50	12.50	00.00	00.00
		MTN-16052020-Money Maker-2		Mild curling and crinkling, as well as minor deformations	TYLCV, ToCV			-	-						
		MTN-16052020-Money Maker-3		Mild curling and crinkling, as well as minor deformations	TYLCV, STV, ToCV			STV	STV						
		MTN-16052020- Roma-4	Leaf (Open field 2)	Plant growth stunting and leaf curling with fruit deformation	TYLCV, STV, ToCV			TYLCV, STV	TYLCV, STV						
		MTN-16052020-Roma-5		Plant growth stunting and leaf infested with mites and whiteflies	TYLCV, TICV, ToCV			-	-						
		MTN-16052020-Naqeeb-6	Leaf (Open field 3)	Leaf curling and yellowing with whiteflies	TYLCV, STV, ToCV, CMV, ToMV			TYLCV	TYLCV						
		MTN-16052020-Naqeeb-7		Leaf curling and yellowing with whiteflies	TYLCV, STV, ToCV, CMV, ToMV			-	-						
		MTN-16052020-Naqeeb-8		No symptoms may be healthy sample	STV			-	-						

Bahawalpur	29°21'15.66"N, 71°41'27.84"E	BWP-21052020-Hybrid-1	Seed/ Leaf (Open field 1)	Severe chlorotic and yellowing symptoms	TYLCV, STV, ToCV, ToMV	50.00	60.00	TY-LCV	TY-LCV	30.00	20.00	00.00	10.00	00.00	00.00
		BWP-21052020-Hybrid-2		Mild chlorotic and yellowing of leaves	TYLCV, STV, ToCV, ToMV			STV	STV						
		BWP-21052020-Hybrid-3		Leaf curling and infested with whiteflies	TYLCV, TICV, ToCV			-	-						
		BWP-21052020-Hybrid-4		Typical yellowing, leaf curling, and deformations	TYLCV, STV, ToCV, CMV, ToMV			STV, TY-LCV, ToCV	STV, TY-LCV, ToCV						
		BWP-21052020-Hybrid-5	Leaf (Open field 2)	Mild mosaic symptoms	ToMV, CMV, ToCV			-	-						
		BWP-21052020-Hybrid-6			STV, ToMV, CMV, TICV			-	-						
		BWP-21052020-1	Leaf (Open field 3)	Stunting, chlorotic, and mild mosaic mottling	TYLCV, ToCV			-	-						
		BWP-21052020-2		Leaf yellowing and curling	STV, TYLCV, ToCV, ToMV, CMV, PVY			STV	STV						
		BWP-21052020-3		Mild mosaic mottling	TYLCV, ToCV, ToMV			-	-						
		BWP-21052020-4			TYLCV, ToCV, ToMV, TICV			-	-						
Lahore	31°31'13.33"N, 74°21'31.49"E	LHR-09062020-1	Leaf (Eastern Army vegetable Farm)	Plant stunted growth and fruit deformation	STV, TYLCV, ToCV, CMV, ToMV	71.42	80.00	-	-	42.85	07.14	21.42	07.14	00.00	00.00
		LHR-09062020-2		Leaf and fruit chlorosis	TYLCV, ToCV			-	-						
		LHR-09062020-3		Leaf yellowing and fruit deformation	STV, TYLCV, ToCV, ToMV			STV, TY-LCV	STV, TY-LCV						
		LHR-09062020-4		Typical yellowing, leaf curling, and deformations	STV, TYLCV, ToCV, ToMV			STV, TY-LCV, ToCV	STV, TY-LCV, ToCV						
		LHR-09062020-5		Typical yellowing, and curling of	TYLCV, ToCV, ToMV										

		emerging leaves									
LHR-09062020-Rutgers			STV, TYLCV		-	-					
LHR-09062020-Hybrid	Seed (Southern Army vegetable Farm)		STV, TYLCV		STV, TYLCV	STV, TYLCV					
LHR-09062020-Early stone		Directly obtained from the seed source	STV, TYLCV		STV	STV					
LHR-09062020-Rio Grande			STV, TYLCV		TYLCV	TYLCV					
LHR-11062020-1		Leaf curling and deformation with severe infestation of whiteflies and mites	ToCV, ToMV, TYLCV, PVY, CMV		TYLCV	TYLCV					
LHR-11062020-2	Leaf (Western vegetable garden 1)	Leaf curling and deformation with severe infestation of whiteflies and mites	STV, ToCV, ToMV, TYLCV, PVY, CMV		-	-					
LHR-11062020-3		Plant stunting, leaf curling and yellowing, and reduced fruit size	STV, ToCV, ToMV, TYLCV		STV	STV					
LHR-11062020-4		No obvious symptoms	STV		-	-					
LHR-11062020-5	Leaf (Western vegetable garden 2)	Typical yellowing, leaf curling, crinkling, and fruit deformations	TYLCV, STV, ToMV, CMV, ToCV		TYLCV, STV, ToCV	TYLCV, STV, ToCV					
Punjab, Pakistan<br>[Total harvested samples= 44, Negative= 18 samples, STV infection= 5 samples, TYLCV infection= 6 samples, STV+TYLCV infection= 7 samples, STV+TYLCV+ToCV infection= 6 samples, STV+TYLCV+ToCV+ToMV infection= 2 sample]<br>* FSD-13022019-Rio Grande sample was also subjected to the Next generation sequencing technology to identify the STV virus from Pakistan.<br>Rate of infections<br>Negative samples= 40.90%<br>STV single infection= 11.36%<br>STV mixed infection= 34.09%<br>STV+TYLCV+ToCV+ToMV infection= 04.54%							11.36	15.90	13.63	00.00	00.00

Abbreviations: AARI, Ayub agricultural research institute; CMV, cucumber mosaic cucumovirus; GPS, global positioning system; PPRI, plant protection research institute; PVY, Potato potyvirus Y; STV, southern tomato amalgavirus; ToCV, tomato chlorosis crinivirus; TICV, tomato infectious chlorosis crinivirus;, ToMV, tomato mosaic tobamovirus, TSWV, tomato spotted wilt orthotospovirus; TYLCV, tomato yellow leaf curl begomovirus; ToYSD, tomato yellow stunt disease; VRI, vegetable research institute. [1] The rate of ToYSD infection (prevalence and incidence) was calculated by the following equations: [a]ToYSD prevalence $= \frac{X}{Y} \times 100$, where X is the number of sample production localities (greenhouses/fields) with visible ToYSD symptoms; Y is the total number of sample production localities observed in a region [26]. [b] ToYSD incidence $= \frac{(N-n)}{N} \times 100$, where N is the total

number of samples under observation, and n is the total number of healthy samples without ToYSD symptoms and viral infections [26].

Table S2. Primer sequences used in this study for the detection of different plant viruses and amplification as well as molecular cloning of STV.

Primer name	Detection of virus	Primer Sequence (5'-3')	Amplicon length	Annealing temperature	Remarks	Reference
**Primers used for the detection of multiple viruses**						
STV-F	Southern tomato amalgavirus	CGTTATCTTAGGCGTCAGCT	448bp	53°C	A fragment covering a conserved region of p42 and RdRp at the position of ribosomal frame shifting.	[8]
STV-R		GGAGTTTGATTGCATCAGCG				
ToCV-16-F	Tomato chlorosis crinivirus	GGTTTGGATTTTGGTACTA-CATTCAGT	466bp	54 °C	An amplicon covering Heat shock protein 70 homolog (HSP70h).	[28,34]
ToCV-481-R		AAACTGCCTG-CATGAAAAGTCTC				
TICV-32-F	Tomato infectious chlorosis crinivirus	TCAGTGCGTACGTTAATGGG	501bp	55 °C	A fragment corresponding to part of the coding sequence of the HSP70h, excluding the phosphate 1 and 2 motif.	[31]
TICV-532-F		CACAGTATACAGCAGCGGCA				
ToMV_527-F	Tomato mosaic tobamovirus	CGAGAGGGGCAACAAACAT	318bp	56 °C	An amplicon covering a portion within the replicase gene.	[29]
ToMV_843-F		ACCTGTCTCCATCTCTTTGG				
TYLCV/IR-F	Tomato yellow leaf curl begomovirus	TAATATTACCKGWKGVCCSC	~500bp	54 °C	Adopted from universal degenerate primers PA and PB corresponding to portion of the intergenic region (IR) and the *AV1* gene of the DNA-A.	[30,32,33]
TYLCV/AV1-R		TGGACYTTRCAWGG-BCCTTCACA (B=C, T or G, K=G or T, R=A or G, S=C or G, V=A, C or G, W=A or T, Y= C or T)				
Tospovirus_ RdRp-F	Tomato spotted wilt orthotospovirus	CCTTTAACAGT(**A**/T/G)GAAAC AT	~800bp	50 °C	Adopted from degenerate primers gL3637 and gL4435c, covering a part of RdRP from L-RNA of orthotospovirus.	[27]
Tospovirus_ RdRp-R		CAT(**A**/T/G)GC(**A**/G)CAAGA(**A**/G)TG(**A**/G)TA(**A**/G)ACAGA				
**Primers designed for the amplification and molecular cloning of STV genome**						
Primer name	**Amplification**	**Primer Sequence (5'-3')**	**Amplicon length**	**Annealing temperature**	**Remarks**	
STV-F1	Amplification of Southern tomato amalgavirus insert part A	ggaagttcatttcatttggagaggGA-TAAATTTAGTAAGCTACCTAGC	1880bp	62 °C	Overlapping pairs of primers, homologous to the vector plasmid (pCB301-2-HDV), were designed for the amplification of STV genome.	
STV-R1		CTTGATCTCTCCGCAG-TATATTTA				
STV-F2	Amplification of Southern tomato amalgavirus insert part B	GTGAGGCGGTTAA-GAAGTTTACAG	1681bp	65 °C		
STV-R2		gaggtggagatgccatgccgacccGAA-GACGCGCTACTCTAATAACAG				
pCB301 backbone-F	Amplification of plasmid pCB301-2μ-HDV	CTGTTATTAGAG-TAGCGCGTCTTCgggtcggcatgg-catctccacctc	7838bp	66 °C	A pair of primers, homologous to STV, were designed to linearize the plasmid (pCB301-2μ-HDV) between the CaMV 35S promoter and the HDRz sequence.	
pCB301 backbone-R		GCTAGGTAGCTTACTA-AATTTATCcctctccaaatgaaatgaacttc c				

Table S3. STV reference isolates used in this study with a detailed description, including accession number, name of isolate, sequence length, country, and host.

No.	Accession	Isolate description	Sequence length	Country	Host
**1**	OK309707	STV, isolate Turkey_Antalya_1	3315	Turkey	*Solanum lycopersicum*
**2**	OK309708	STV, isolate Turkey_Antalya_6	3315	Turkey	*S. lycopersicum*
**3**	OK309709	STV, isolate Turkey_Balikesir_2	3315	Turkey	*S. lycopersicum*
**4**	OK309710	STV, isolate Turkey_Balikesir_5	3315	Turkey	*S. lycopersicum*
**5**	OK309711	STV, isolate Turkey_Canakkale_42	3315	Turkey	*S. lycopersicum*
**6**	OK309712	STV, isolate Turkey_Manisa_12	3315	Turkey	*S. lycopersicum*
**7**	OK309713	STV, isolate Turkey_Manisa_8	3315	Turkey	*S. lycopersicum*

**8**	OK309714	STV, isolate Turkey_Canakkale_16	3315	Turkey	*S. lycopersicum*
**9**	OK309715	STV, isolate Turkey_Edirne_32	3315	Turkey	*S. lycopersicum*
**10**	OK309716	STV, isolate Turkey_Tekirdag_29	3315	Turkey	*S. lycopersicum*
**11**	OK309717	STV, isolate Turkey_Izmir_60	3315	Turkey	*S. lycopersicum*
**12**	OK309718	STV, isolate Turkey_Izmir_63	3315	Turkey	*S. lycopersicum*
**13**	OK309719	STV, isolate Turkey_Izmir_65	3315	Turkey	*S. lycopersicum*
**14**	OK309720	STV, isolate Turkey_Bursa_52	3315	Turkey	*S. lycopersicum*
**15**	OK309721	STV, isolate Turkey_Bursa_56	3315	Turkey	*S. lycopersicum*
**16**	LC429302	STV, M82 genomic RNA	3376	Japan	*S. lycopersicum*
**17**	MW266062	STV, isolate 21807453	3393	United Kingdom	*S. lycopersicum*
**18**	MW012413	STV, isolate DDT	3398	Vietnam	*S. lycopersicum*
**19**	MW012412	STV, isolate DCT	3411	Vietnam	*S. lycopersicum*
**20**	MK610257	STV, isolate Canada	3414	Canada	*S. lycopersicum*
**21**	MW012411	STV, isolate DTT	3415	Vietnam	*S. lycopersicum*
**22**	MG808383	STV, isolate STV-MG	3416	Brazil	*S. lycopersicum*
**23**	MW012410	STV, isolate GLT	3425	Vietnam	*S. lycopersicum*
**24**	KJ174690	STV, isolate GCN06	3426	Spain	*S. lycopersicum*
**25**	KT438549	STV, isolate CN-12	3430	China	*S. lycopersicum*
**26**	EF442780	STV, isolate Mexico-1	3433	Mexico	*S. lycopersicum*
**27**	EU413670	STV, isolate MS-7	3433	USA	N/A
**28**	KT634055	STV, isolate BD-13	3433	Bangladesh	*S. lycopersicum*
**29**	KT852573	STV, isolate NC12-03-08	3433	USA	*S. lycopersicum*
**30**	KX949574	STV, isolate Florida	3433	USA	*S. lycopersicum*
**31**	KY228384	STV, isolate XJ-p	3433	China	*S. lycopersicum*
**32**	KY810783	STV, strain FERA_160205	3433	United Kingdom	*S. lycopersicum*
**33**	LC270272	STV, genomic RNA , isolate: Gimcheon	3433	South Korea	*S. lycopersicum*
**34**	LC487710	STV, Thailand genomic RNA	3433	Thailand	*Capsicum annuum*
**35**	MF422617	STV, isolate CH_bpo161	3433	Switzerland	*S. lycopersicum*
**36**	MF422618	STV, isolate CH_bpo163	3433	Switzerland	*S. lycopersicum*
**37**	MN095716	STV, isolate Antioquia/May5	3433	Colombia	*S. lycopersicum*
**38**	MT051992	STV, isolate STV_Panama	3433	Panama	*S. lycopersicum*
**39**	MT066231	STV, isolate Pk	3437	Pakistan	*S. lycopersicum*
**40**	MT269808	STV, isolate 232-11	3433	Serbia	*S. lycopersicum*
**41**	MT269809	STV, isolate 323-12	3433	Serbia	*S. lycopersicum*
**42**	MW520860	STV, isolate RE-RE1375-Grand Tampon-2018	3433	Reunion	*S. lycopersicum*
**43**	OP548652	STV, isolate Tongzhou, Beijing	3437	China	*S. lycopersicum*
**44**	OP548653	STV, Isolate Fangshan, Beijing	3437	China	*S. lycopersicum*

Reference:

8. Sabanadzovic, S.; Valverde, R.A.; Brown, J.K.; Martin, R.R.; Tzanetakis, I.E. Southern tomato virus: The link between the families Totiviridae and Partitiviridae. *Virus Res*. 2009, 140, 130–137.

26. Saokham, K.; Hemniam, N.; Roekwan, S.; Hunsawattanakul, S.; Thawinampan, J.; Siriwan, W. Survey and molecular detection of Sri Lankan cassava mosaic virus in Thailand. *PLoS ONE* 2021, 16, e0252846.

27. Chu, F.H.; Chao, C.H.; Chung, M.H.; Chen, C.C.; Yeh, S.D. Completion of the genome sequence of Watermelon silver mottle virus and utilization of degenerate primers for detecting tospoviruses in five serogroups. *Phytopathology* 2001, 91, 361–368.

28. Dovas, C.; Katis, N.; Avgelis, A. Multiplex detection of criniviruses associated with epidemics of a yellowing disease of tomato in Greece. *Plant Dis*. 2002, 86, 1345–1349.

29. Kumar, S.; Udaya Shankar, A.; Nayaka, S.; Lund, O.; Prakash, H. Detection of Tobacco mosaic virus and Tomato mosaic virus in pepper and tomato by multiplex RT–PCR. *Lett. Appl. Microbiol*. 2011, 53, 359–363.

30. Li, Z.; Zhou, X.; Zhang, X.; Xie, Y. Molecular characterization of tomato-infecting begomoviruses in Yunnan, China. *Arch. Virol*. 2004, 149, 1721–1732.

31. Vaira, A.; Accotto, G.; Vecchiati, M.; Bragaloni, M. Tomato infectious chlorosis virus causes leaf yellowing and reddening of tomato in Italy. *Phytoparasitica* 2002, 30, 290–294.
32. Xie, Y.; Jiao, X.; Zhou, X.; Liu, H.; Ni, Y.; Wu, J. Highly sensitive serological methods for detecting tomato yellow leaf curl virus in tomato plants and whiteflies. *Virol. J*. 2013, 10, 142.
33. Xie, Y.; Zhou, X.; Zhang, Z.; Qi, Y. Tobacco curly shoot virus isolated in Yunnan is a distinct species of Begomovirus. *Chin. Sci. Bull*. 2002, 47, 199–201.
34. Zhao, R.; Wang, R.; Wang, N.; Fan, Z.; Zhou, T.; Shi, Y.; Chai, M. First report of Tomato chlorosis virus in China. *Plant Dis*. 2013, 97, 1123–1123.
